# Comparative Clinical Outcomes of Needle Aspiration and Incision & Drainage in Peritonsillar Abscess

**DOI:** 10.3390/jcm15062347

**Published:** 2026-03-19

**Authors:** Melih Alpay, Nesibe Gül Yüksel Aslier, Mert Anıl Danişman, Fuat Bulut, Betul Agirgol, Hakkı Caner Inan

**Affiliations:** Department of Otolaryngology, Bursa High Specialty Training and Research Hospital, University of Health Sciences, Bursa 16310, Türkiye; nesibe.gul.yuksel@gmail.com (N.G.Y.A.); mertanild@gmail.com (M.A.D.); bulutfuat40@yahoo.com (F.B.); betularisoy@gmail.com (B.A.); hakkicanerinan@gmail.com (H.C.I.)

**Keywords:** drainage, incision, needle aspiration, peritonsillar abscess, tonsillitis

## Abstract

**Background:** Peritonsillar abscess (PTA) is a common deep neck infection associated with significant healthcare resource utilization. Needle aspiration (NA) and/or incision & drainage (ID) are the most frequently used treatment modalities; however, their comparative clinical outcomes remain controversial. **Methods:** This retrospective cohort study included 185 adult patients hospitalized with a diagnosis of PTA between January 2018 and January 2026 at a tertiary care center. Patients aged ≥18 years who underwent NA or ID were included. Demographic characteristics, clinical findings, laboratory parameters at admission, treatment-related variables (type and number of interventions, drainage volume, adjunctive corticosteroid use), microbiological results, and length of hospital stay were recorded. Patients were categorized according to the drainage method performed (NA vs. ID). **Results:** A total of 185 patients were included, of whom 87 (47%) underwent NA and 98 (53%) underwent ID. There were no significant differences between groups in age, sex, or length of hospital stay (*p* > 0.05). CRP levels and lymphocyte counts were significantly higher in the NA group (*p* = 0.028 and *p* = 0.009, respectively), whereas drainage volume and number of interventions were significantly higher in the ID group (*p* < 0.001 and *p* = 0.005, respectively). Successful resolution on the first attempt was more frequent in the ID group (89.8% vs. 75.9%, *p* = 0.011), while steroid use was more common in the NA group (16.1% vs. 2.0%, *p* = 0.001). In multivariate analysis, drainage volume, first-attempt success, and corticosteroid use were independently associated with the choice of drainage method (*p* < 0.05). **Conclusions:** Both NA and ID are effective treatment options for PTA; however, ID was associated with higher first-attempt success and greater drainage volume. Indeed, NA was more frequently accompanied by steroid therapy. These findings suggest that treatment modality selection may influence short-term clinical outcomes in patients with PTA.

## 1. Introduction

Peritonsillar abscess (PTA) is one of the most common conditions in otorhinolaryngology requiring hospitalization and represents a localized collection of pus between the fibrous capsule of the tonsil and the superior constrictor muscle [1]. Although PTA is traditionally considered a suppurative complication of acute tonsillitis, infections of the Weber glands located at the superior pole of the tonsil have also been implicated in its pathogenesis [2]. PTA is the most frequent deep neck space infection, with an annual incidence of approximately 30 cases per 100,000 individuals in the United States [3] and 19 cases per 100,000 individuals in Germany [4]. Patients typically present with fever, severe sore throat, and muffled or unintelligible speech and may also exhibit trismus, reactive jugulodigastric lymphadenopathy, and ipsilateral soft palate edema with erythema [5]. Delayed or inadequate treatment of PTA may lead to serious complications, including parapharyngeal abscess, mediastinitis, necrotizing fasciitis, and internal jugular vein thrombosis (Lemierre’s syndrome) [6]. In addition, PTA may result in life-threatening conditions such as airway obstruction and sepsis [7]. Therefore, prompt diagnosis and effective management are crucial.

Surgical intervention remains the cornerstone of PTA treatment, with reported success rates exceeding 90%. The first approach primarily consists of needle aspiration (NA) or incision and drainage (ID) [8]. Despite their widespread use, the optimal choice between these two techniques remains controversial.

The objective of this retrospective cohort study was to compare the clinical outcomes and treatment-related variables of NA versus ID in adult patients hospitalized with PTA. Specifically, the study aimed to evaluate differences in demographics, laboratory and clinical characteristics, and success rates between the two surgical modalities.

## 2. Materials and Methods

This study was approved by the Ethics Committee of Bursa Specialized Teaching Hospital at Health Sciences University with the Committee Decision No. 2011-KAEK-25 2023/07-07 and conducted at a tertiary teaching hospital.

Adult patients hospitalized with the diagnosis of PTA between January 2018 and January 2026 were evaluated.

A total of 185 consecutive patients who underwent either NA or ID were included after the inclusion and exclusion criteria described below were implemented. With group sizes of 87 and 98, a two-tailed α of 0.05, and 80% statistical power, the study is adequately powered to detect a small-to-moderate effect size (Cohen’s d ≈ 0.41).

Inclusion criteria:

1. Patients ≥ 18 years.
2. Patients who had undergone the first PTA management at our hospital.

Exclusion criteria:

1. Patients younger than 18 years;
2. Patients who had undergone previous drainage at another healthcare facility,
3. Patients with abscesses secondary to malignancy or prior surgery;
4. Patients who had incomplete hospital records.

All patients received inpatient treatment and were discharged after clinical stabilization, defined as resolution of fever, improvement in physical examination findings, restoration of oral intake, and decline in inflammatory markers. Patients were categorized into NA and ID groups according to the drainage procedure performed. NA was performed under local anesthesia using a 24-gauge needle inserted at the point of maximal fluctuance. Negative pressure was applied to aspirate purulent material until no further drainage was obtained. ID was carried out under local anesthesia by making a small mucosal incision over the most prominent and fluctuant area, followed by blunt dissection to break loculations and allow complete evacuation of pus. Both residents and attending otolaryngologists performed the procedures in accordance with institutional supervision protocols. In our center, residents carry out procedures under either direct or indirect supervision by attending physicians. The majority of procedures were performed in the emergency ORL room. Patients requiring airway monitoring or further intervention were managed in the operating room.

Data were extracted from electronic medical records. The recorded variables included demographic characteristics (age and sex), smoking status, comorbidities, presenting symptoms and physical examination findings, imaging modalities and involvement of adjacent deep neck spaces, presence of lymphadenopathy, and laboratory parameters obtained at admission, including white blood cell count, absolute neutrophil and lymphocyte counts, and C-reactive protein (CRP) levels. Computed tomography (CT) imaging was not routinely performed. It was reserved for selected cases, including suspicion of deep neck space involvement, atypical clinical presentation, diagnostic uncertainty, or inadequate response to initial treatment. The decision to perform needle NA or ID was primarily based on clinical examination findings rather than CT results.

Treatment-related variables comprised the type of intervention (NA or ID), number of interventions, volume of drained purulent material, adjunctive systemic corticosteroid use, and microbiological culture results. Clinical outcomes, including length of hospital stay and success at the first admission, were also documented. Treatment success was defined as clinical improvement without the need for additional surgical intervention during hospitalization.

All patients received empirical intravenous antibiotics on admission. Antibiotic regimens included ampicillin–sulbactam, metronidazole, clindamycin, clarithromycin, or ceftriaxone, administered as monotherapy or combination therapy according to physician preference and allergy history. Antibiotic therapy was guided primarily by clinical response rather than culture results. Due to the high prevalence of prior antibiotic use and the polymicrobial nature of peritonsillar abscesses, treatment was continued according to institutional protocols even when cultures were negative.

Intravenous hydration with isotonic saline and 5% dextrose was provided until adequate oral intake was achieved. Adjunctive systemic corticosteroids were administered when clinically indicated. Patients were discharged after resolution of symptoms and clinical stabilization.

Statistical analyses were performed using IBM SPSS Statistics version 24.0 (IBM Corp., Armonk, NY, USA). Normality was assessed with the Shapiro–Wilk test. Continuous variables were presented as mean ± standard deviation or median (minimum–maximum), as appropriate, and categorical variables as frequencies and percentages. Group comparisons between NA and ID were conducted using the independent-sample *t*-test or Mann–Whitney U test for continuous variables and the chi-square or Fisher–Freeman–Halton test for categorical variables. Variables significant in univariate analysis were entered into a binary logistic regression model. A two-tailed *p*-value < 0.05 was considered statistically significant.

## 3. Results

The study included 185 patients who received inpatient treatment for PTA. Of these, 111 (60%) were male, and 74 (40%) were female. The median age of the patients was 32 years (range: 18–75). Among the participants, 51 (27.6%) were smokers. The most common comorbidities were hypertension (5.3%), diabetes (3.2%), and asthma (2.2%). Detailed demographic and clinical characteristics are summarized in Table 1. Clinical signs and symptoms at admission included pain (100%), peritonsillar swelling (100%), uvula deviation (33.5%), trismus (28.6%), and dysphagia (26.5%). CT imaging was performed in 76 (41.1%) patients to support the diagnosis. Regarding the location, the right tonsil was affected in 53.5% of cases and the left in 46.5%.

Patients were divided into two groups based on the drainage method: NA (n = 87, 47%) and ID (n = 98, 53%). Univariate analysis revealed no significant differences between the groups regarding age (*p* = 0.476), sex (*p* = 0.417), or length of hospital stay (*p* = 0.638). However, several laboratory and clinical parameters showed significant differences (Table 1). CRP levels were significantly higher in the NA group (median: 152.00) compared to the ID group (median: 123.50; *p* = 0.028). Similarly, the mean lymphocyte count was significantly higher in the NA group (2334 ± 741) than in the ID group (2044 ± 743; *p* = 0.009). The drainage volume was significantly higher in the ID group (median: 6.00 cc) compared to the NA group (median: 3.00 cc; *p* < 0.001). Furthermore, the number of interventions was significantly higher in the ID group (*p* = 0.005). The average length of hospital stay was 5.3 days in the NA group and 5.45 days in the ID group. No statistically significant differences were found between the two groups in the length of hospital stay (*p* = 0.638).

Abscess cultures were positive in 51 (27.6%) patients, while 134 (72.4%) patients showed no growth. Among the isolated microorganisms, *Streptococcus pyogenes* was the most frequently identified pathogen (n = 22, 11.9% of the total cohort), followed by other *Streptococcus* species. The complete distribution of isolated bacteria is presented in Table 2.

Binary logistic regression analysis was performed to identify independent predictors of the drainage approach (Table 3). The volume of drained pus was significantly higher in the ID group compared to the needle aspiration group (*p* < 0.001). The rate of successful resolution on the first attempt was significantly higher in the ID group (89.8%) compared to the NA group (75.9%) (*p* = 0.011). Notably, corticosteroid usage was significantly more frequent in the needle aspiration group (16.1%) compared to the incision and drainage group (2.0%) (*p* = 0.001).

## 4. Discussion

This study evaluated clinical and laboratory outcomes in 185 patients hospitalized with PTA. Our results highlight that while both NA and ID are effective, ID provides superior initial source control, whereas NA is more frequently associated with adjunctive corticosteroid use. A primary finding of this study was the significantly higher drainage volume and first-attempt success rate in the ID group. This is consistent with the research of Viljoen and Loock, which identified the volume of pus evacuated during the initial procedure as a strong predictor of the need for subsequent re-aspirations [9]. In our study, the greater volume removed via ID likely explains why this group achieved a higher rate of successful resolution at the first attempt compared to the NA group.

The optimal choice between NA and ID remains a subject of debate in current research. Mansour et al. reported that patients undergoing ID experienced shorter hospital stays [10], whereas Johnson et al. found no significant differences between NA and ID regarding hospital stay or recurrence [11]. Our findings align with those of Johnson et al., as we observed no statistically significant difference in hospital stay.

The multivariate analysis demonstrated that drainage volume and first-attempt success independently influenced the selection of the drainage method. This finding suggests that procedural choice in clinical decision-making is not arbitrary but rather guided by objective parameters such as infection severity and the initial response to treatment.

Corticosteroids are often utilized to manage symptoms like trismus and severe dysphagia, which were prevalent in our study [12,13]. An interesting clinical observation was that corticosteroid usage was significantly more frequent in the NA group (16.1%) than in the ID group (2.0%). This may reflect a clinical strategy to compensate for potentially less efficient drainage in the NA group by utilizing the anti-inflammatory effects of corticosteroids to reduce edema and pain. While corticosteroids are known to improve symptomatic relief in PTA, their use in our study was independently associated with the NA approach in the logistic regression model.

Microbiologically, *Streptococcus pyogenes* was the most frequently isolated pathogen (11.9%), mirroring the findings of Saar et al. and Slouka et al. [5,14]. The high rate of culture-negative results (72.4%) in our study is a common challenge in PTA management, often attributed to antibiotic use prior to hospital admission, as also noted by Mazur et al. [15]. In our cohort, culture negativity did not lead to discontinuation of antibiotics. This approach reflects both the frequent pre-admission antibiotic exposure among patients and the polymicrobial characteristics of peritonsillar abscesses. Therefore, clinical response remained the primary criterion for guiding therapy, in accordance with the institutional approach.

Regarding patient demographics, the observed male predominance (60%) is consistent with the findings of Schwarz et al. [16]. Furthermore, comorbidities such as type 2 diabetes mellitus (T2DM), though present in a small percentage of our patients, are critical factors; as Wu et al. demonstrated, T2DM significantly increases susceptibility to PTA [17].

Despite notable strengths, including a robust sample size, multivariate statistical approach, and clinically meaningful findings, there are some limitations, including its retrospective design and inpatient setting. Our sample was composed of patients requiring hospitalization and may not be directly generalizable to all cases of peritonsillar abscess treated in outpatient settings. However, we believe that this specific patient population is clinically very important, as hospitalized cases often represent more severe or complicated presentations, for which an optimal drainage strategy remains an important question. The non-randomized nature of the study represents an important limitation. As this was a retrospective analysis reflecting real-world clinical practice in a tertiary referral center, random allocation was not feasible, and selection bias might have been an issue. The choice of drainage technique was based on physician preference, which may introduce another selection bias.

Nonetheless, the results support the use of ID as a highly reliable first-line intervention for inpatient management, particularly when larger abscess volumes are suspected.

## 5. Conclusions

Both NA and ID are effective surgical approaches for PTA. In our cohort, ID was associated with higher initial treatment success and greater drainage volume, whereas NA more frequently required adjunctive systemic corticosteroid therapy. These observations suggest that the selected drainage technique may influence short-term outcomes in hospitalized patients. Further prospective studies using standardized treatment protocols are warranted to clarify the optimal management strategy and to better define patient-specific factors that may guide individualized clinical decision-making. Future research should ideally include randomized controlled trials directly comparing NA and ID, which would help minimize selection bias and enable stronger causal inferences.

## Figures and Tables

**Table 1:** Univariate analysis of variables according to drainage method.

	Overall	Needle Aspiration	Incision and Drainage	
	(n = 185)	(n = 87)	(n = 98)	*p*
Age	32.00 (18–75)	33.00 (18–73)	30.50 (19–75)	0.476
Median (Min–Max)				
Sex n (%)				0.417
Female	74 (40%)	32 (43.2%)	42 (56.8%)	
Male	111 (60%)	56 (50.5%)	55 (49.5%)	
Smoking; n (%)				0.873
Smoker	51 (27.6%)	23 (26.4%)	28 (28.6%)	
Non-Smoker	134 (72.4%)	64 (73.6%)	70 (71.4%)	
Comorbidities n (%)				
Asthma	4 (2.2%)	0 (0.0%)	4 (4.1%)	0.076
Hypertension	15 (8.1%)	5 (5.7%)	10 (10.2%)	0.295
Diabetes	6 (3.2%)	2 (2.3%)	4 (4.1%)	0.686
Symptoms and Signs n (%)				
Pain	185 (100.0%)	87 (100.0%)	98 (100.0%)	>0.999
Dysphagia	49 (26.5%)	23 (26.4%)	26 (26.5%)	>0.999
Trismus	53 (28.6%)	25 (28.7%)	28 (28.6%)	0.554
Peritonsillar Swelling	185 (100.0%)	87 (100.0%)	98 (100.0%)	>0.999
Uvula Deviation	62 (33.5%)	24 (27.6%)	38 (38.8%)	0.120
Imaging n (%)				0.329
None	109 (58.9%)	48 (55.2%)	61 (62.2%)	
CT	76 (41.1%)	39 (44.8%)	37 (37.8%)	
Involved Area n (%)				
Parapharyngeal	5 (2.7%)	3 (3.4%)	2 (2.0%)	0.667
Retropharyngeal	2 (1.1%)	1 (1.1%)	1 (1.0%)	>0.999
Abscess Culture n (%)				0.162
Positive	51 (27.6%)	21 (24.1%)	30 (30.6%)	
Negative	134 (72.4%)	66 (75.9%)	68 (69.4%)	
CRP				0.028
Median (Min–Max)	143.00 (4–998)	152.00 (9–998)	123.50 (4–979)	
WBC				0.132
Mean ± SD	14,783 ± 4364	15,296 ± 4652	14,328 ± 4061	
Neutrophil				0.544
Mean ± SD	11,551 ± 3999	11,741 ± 4458	11,382 ± 3557	
Lymphocyte				0.009
Mean ± SD	2180 ± 755	2334 ± 741	2044 ± 743	
Neutrophil Lymphocyte Ratio				0.066
Median (Min–Max)	5.30 (0.84–19.08)	4.82 (0.84–19.08)	5.55 (0.91–15.70)	
Length of Stay in Hospital				0.638
Median (Min–Max)	5.00 (2–14)	5.00 (2–9)	5.00 (2–14)	
Lymphadenopathy				0.868
Median (Min–Max)	0.00 (0–4)	0.00 (0–4)	0.00 (0–3)	
Drainage Volume				<0.001
Median (Min–Max)	5.00 (0–22)	3.00 (0–15)	6.00 (1–22)	
Number of Interventions				0.005
Median (Min–Max)	2.00 (1–5)	1.00 (1–4)	2.00 (1–5)	
Long Diameter				0.680
Mean ± SD	18.88 ± 5.29	19.13 ± 5.69	18.62 ± 4.90	
Short Diameter				0.794
Median (Min–Max)	15.00 (7–31)	15.00 (7–31)	14.00 (9–23)	

Variables are presented as mean ± standard deviation (SD) or median (minimum–maximum), and number (%), according to normality and statistical analyses.

**Table 2:** Microbiological distribution of pathogens isolated from abscess cultures.

Bacteria Culture Test Result	n	%
*Streptococcus pyogenes*	22	11.9
*Streptococcus* spp.	9	4.9
*Streptococcus mitis*	5	2.7
*Streptococcus agalactiae*	2	1.1
*Staphylococcus aureus*	2	1.1
*Streptococcus anginosus*	2	1.1
*Streptococcus pneumonia*	2	1.1
*Streptococcus constellatus*	1	0.5
*Streptococcus paranguinis*	1	0.5
MSSA	1	0.5
MRCNS	1	0.5
*Micrococcus lylae*	1	0.5
*Klebsiella pneumoniae*	1	0.5
*Enterococcus* spp.	1	0.5
Culture Negative	134	72.4

MRSA: methicillin-sensitive *Staphylococcus aureus*; MRCNS: methicillin-resistant coagulase-negative *Staphylococci*.

**Table 3:** The results of binary logistic regression analysis for multivariate analysis of significant variables and drainage approach.

	Binary Logistic Regression Analysis (Model: Significance ≤ 0.001, Nagelkerke R^2^ = 0.380, Predicted Percentage Correct = 73.5%)				
Variables	Wald	95% CI Lower	95% CI Upper	OR	*p* Value
CRP	1.154	0.997	1.001	0.999	0.283
Lymphocyte count	2.533	0.999	1.000	1.000	0.112
Drainage volume (cc)	12.756	1.125	1.499	1.299	<0.001
Success in first attempt	6.499	0.047	0.670	0.177	0.011
Steroid treatment	5.495	1.464	71.358	10.219	0.019

## Data Availability

The data that support the findings of this study are available from the corresponding author upon reasonable request.
